# Lateral meniscus injuries have more impact on pivot shift than anterolateral complex injuries in anterior cruciate ligament‐injured knees

**DOI:** 10.1002/ksa.70027

**Published:** 2025-09-09

**Authors:** Lukas Willinger, Armin Runer, Romed P. Vieider, Andrea Achtnich, Julian Mehl, Sebastian Siebenlist, Philipp Winkler

**Affiliations:** ^1^ Department of Sport Orthopaedics, TUM University Clinic, Klinikum rechts der Isar Technical University Munich Munich Germany; ^2^ Department for Orthopaedics and Traumatology, Kepler University Hospital GmbH Johannes Kepler University Linz Linz Austria

**Keywords:** ACL reconstruction, anterolateral complex, anterolateral rotatory knee instability, Kaplan fibres, lateral meniscus, PIVOT application

## Abstract

**Purpose:**

The purpose of this prospective study was to investigate the effect of Kaplan fibres (KF), anterolateral ligament (ALL) and lateral meniscus (LM) injuries on preoperative anterolateral rotational instability (ALRI) in anterior cruciate ligament (ACL)‐injured knees. It was hypothesised that injuries to the ALC (i.e., KF and ALL) and to the LM would increase the preoperative anterolateral rotatory knee instability (ALRI) quantified by the pivot shift (PS) test.

**Methods:**

A prospective registry study was conducted and patients who underwent primary unilateral ACL reconstruction were included. The PS test was preoperatively performed and quantified using the PIVOT iPad application. The anterior translation of the lateral tibia plateau (ATLT) was measured and the side‐to‐side difference (SSD) was calculated. The PS test was additionally graded according to IKDC criteria. Injuries to the KF, ALL and LM were diagnosed on acute preoperative magnetic resonance imaging (MRI) scans. Student's *t*‐test was used to compare means and binary regression analysis was used to calculate odds ratio (OR). Statistical significance was set to *p* < 0.05.

**Results:**

One hundred and twenty‐four patients with a mean age of 29.9 ± 11.2 years were included in this study (61% male, 61% right knees). Patients with a LM injury showed a higher likelihood of having a high‐grade PS (odds ratio [OR] = 2.5, *p* = 0.045). Complete radial meniscus tears of the LM significantly increased the ATLT in the quantified PS test compared to patients with an intact meniscus (*p* < 0.05). Injuries to the ALL, to the KF or a combination of both were not associated with high‐grade PS or an increased ATLT.

**Conclusion:**

Concomitant LM tears were associated with a clinical high‐grade PS. Complete radial meniscus tears of the lateral meniscus significantly increased the ATLT compared to intact meniscus. Injuries to the ALL or to the KF were not associated with high‐grade PS or an increased ATLT. This study provides new clinical evidence that concomitant LM injuries contributes to increased ALRI, highlighting the importance of addressing these structures during ACL reconstruction to optimise rotational instability.

**Level of Evidence:**

Level III diagnostic studies.

**Abbreviations**
ACL: anterior cruciate ligament
ALC: anterolateral complex
ALL: anterolateral ligament
ALRI: anterolateral rotational instability
ATLT: anterior translation of the lateral tibia plateau
BMI: body mass index
IKDC: International Knee Documentation Committee
ITB: iliotibial band
KF: Kaplan fibres
LM: lateral meniscus
MRI: magnetic resonance imaging
OR: odds ratio
PS: pivot shift

## INTRODUCTION

The anterior cruciate ligament (ACL) is the primary stabiliser against anterior tibial translation and a secondary stabiliser for internal rotation of the knee, with its injury being one of the most common ligamentous disruptions in athletes and active individuals. The pivot‐shift test (PS), which assesses rotatory instability of the knee, is a cornerstone in evaluating the clinical and functional impact of ACL injuries [3, 25]. While traditionally a qualitative assessment was used, advances in technology have enabled the quantification of the PS, offering greater objectivity in characterising rotatory instability of the knee [20].

Concomitant meniscal tears and disruptions to the anterolateral complex (ALC) including the anterolateral ligament (ALL) and the Kaplan fibres (KF) frequently accompany ACL injuries and influence the biomechanical behaviour of the knee [4, 23, 24, 34, 37, 46]. These secondary injuries have the potential to exacerbate instability, affect surgical planning and postoperative outcomes. However, the precise role of concomitant injuries in influencing preoperative PS severity remains incompletely understood.

Current evidence suggests that meniscal integrity, particularly of the lateral meniscus (LM), plays a pivotal role in controlling anterolateral rotatory knee stability [23, 34, 39]. Likewise, damage to the ALC with its anatomical structures may synergistically increase knee laxity [1, 9]. Quantifying these effects could enhance the understanding of injury patterns and guide individualised treatment strategies. Nevertheless, the interaction between ACL insufficiency and associated injuries in shaping preoperative PS metrics has not been clinically examined conclusively.

This study aimed to evaluate the effect of concomitant lateral compartment injuries on preoperative clinical and quantitative PS in acute primary ACL‐injured knees. It was hypothesised that injuries to the ALC (i.e., KF and ALL) and to the LM would increase the preoperative anterolateral rotatory knee instability (ALRI) quantified by the PS test.

## MATERIALS AND METHODS

This was a prospective registry study approved by the Ethics Committee of the Technical University of Munich (No.: 198/21 S‐KK). Patients gave their informed consent before enrollment in the study.

Patients aged between 16 and 55 years who underwent unilateral primary ACL reconstruction between January 2021 and September 2024 were recorded in the registry. Patients were included in the final analysis when preoperative magnetic resonance imaging (MRI) scans were available and performed within 28 days after ACL injury and if there was no history of contralateral knee injury or surgery. Exclusion criteria comprised skin disorders in the area of the knee joint, a body mass index (BMI) of ≥35 kg/m^2^ due to possible increased skin mobility or difficulties in identifying anatomical landmarks, and any injuries to the medial and/or lateral collateral ligaments with at least grade II° instability. Patients with a concomitant medial or lateral bucket handle tear in the preoperative MRI scans were also excluded due to the potential effect on knee kinematics (e.g., blocking).

### Clinical examination of antero‐lateral rotatory knee laxity

Clinical examination of antero‐lateral rotatory knee laxity was performed by one of three independent investigators (L.W., A.R. and P.W.W.). The investigators were trained in using the PIVOT iPad application (Version 1.05, University of Pittsburgh, USA) before starting to test patients' PS in the operation room. The injured index and the healthy contralateral knees were investigated preoperatively with the patient under general anaesthesia to exclude any influence of patients' apprehension or muscle tension.

**Figure 1:**
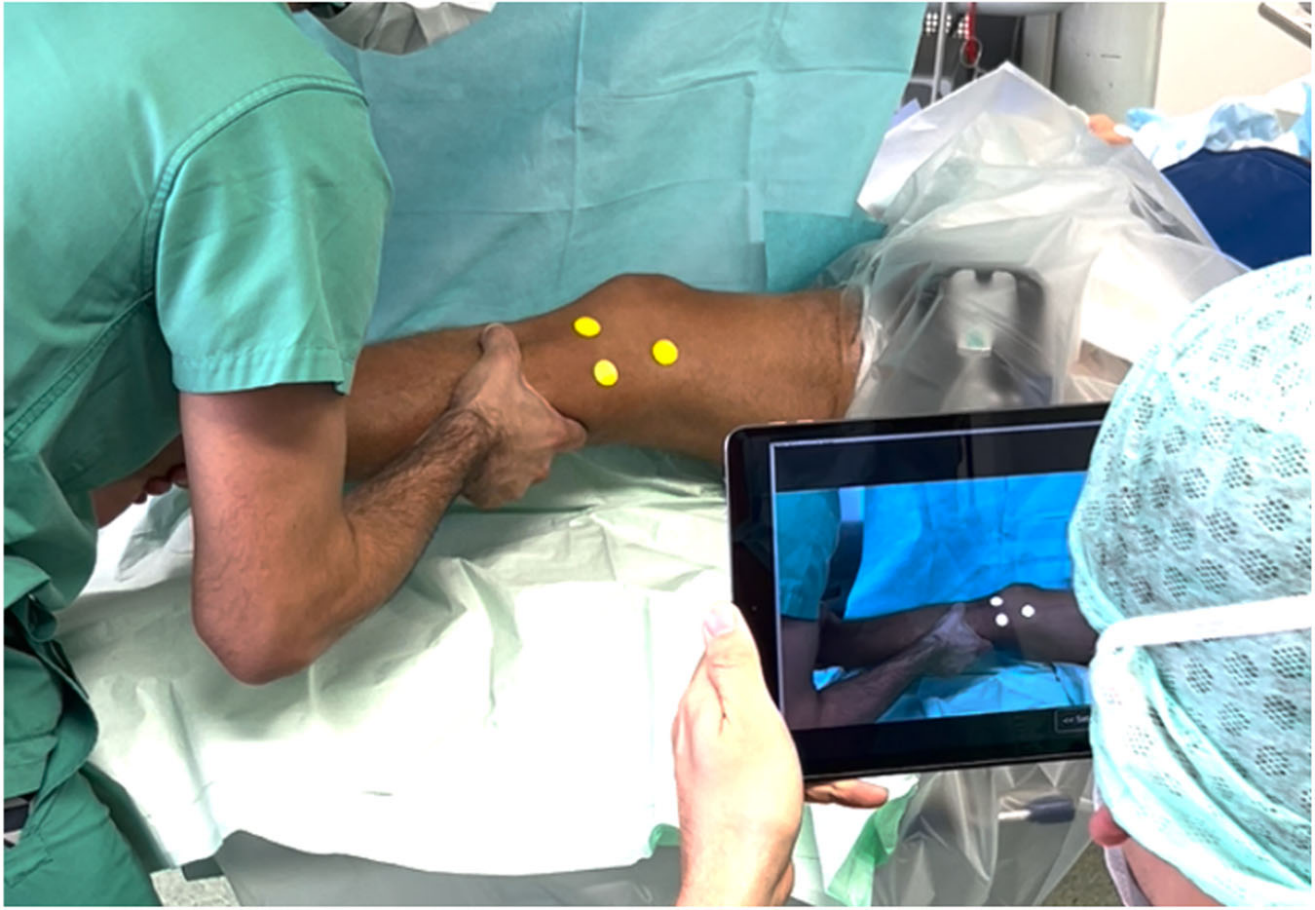
Intraoperative setup and measurement of the pivot shift using the PIVOT iPad application. The markers are placed in the lateral femoral epicondyle, Gerdy's tubercle and 3 cm posterior to it. The pivot shift test is performed and recorded by the iPad camera.

Participants were placed supine on the operating table with the examined knee held by the investigator. Three yellow adhesive surface markers with a diameter of 19 mm were placed on predefined landmarks on the lateral side of the knee as following: Marker 1, on the lateral femoral epicondyle; Marker 2, directly on the osseous prominence of Gerdy's tubercle; Marker 3, 8 cm posterior to Marker 2 (Figure 1). The PS test was used to assess antero‐lateral rotatory knee instability, with an independent observer recording each knee examination using the PIVOT iPad app (version 1.05, University of Pittsburgh) [21, 22]. This freely available app, designed for commercial iPads, quantifies lateral knee compartment translation during the PS test using a validated image analysis software. It demonstrates strong correlation with 3D bony motion and good intra‐ and interrater agreement, with an average measurement error under 6% [30]. The PIVOT iPad app has been validated and used in many previous studies [31, 33, 34, 38, 47].

In this study, the PIVOT iPad app measured relative motion between a marker on the lateral femoral epicondyle (Marker 1) and two tibial markers (Markers 2 and 3), defined as anterior translation of the lateral tibial plateau (ATLT). To ensure accuracy, recordings were made with the iPad positioned 100 cm (75–150 cm) away, perpendicular to the markers, against a monotone background to reduce interference [21]. After recording, the ATLT curve was analysed on the app's output screen, with appropriate data points selected for precise measurements. All data from the PIVOT iPad application were collected by one observer (L.W.).

In addition to the quantification, the PS test was graded according to the classification of the International Knee Documentation Committee (IKDC) in grade I: grade (glide), II (clunk) and grade III (gross). For further analysis, it was divided into low‐grade pivot shift (negative and grade I) and high‐grade pivot shift (grade II and III).

### Evaluation of preoperative MRI scans

**Figure 2:**
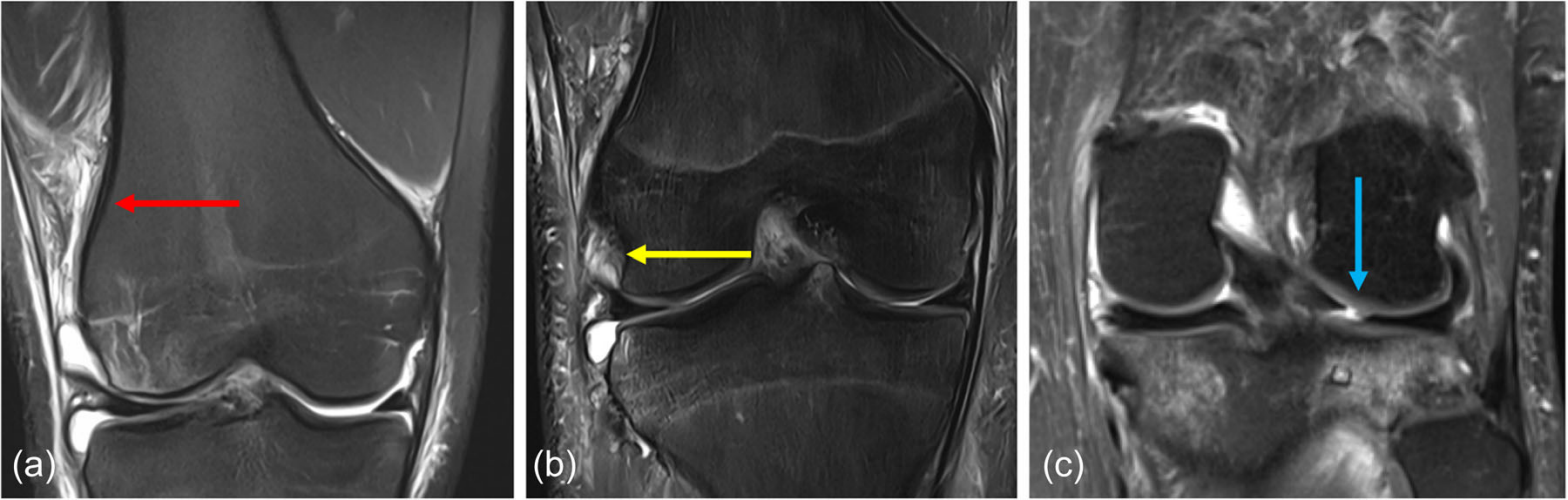
Preoperative magnetic resonance images were analysed to assess the integrity of anterolateral structures and the lateral meniscus. (a) Red arrow: Avulsion of the proximal and distal Kaplan fibres with soft tissue oedema. (b) Yellow arrow: Injured anterolateral capsule and ligament. (c) Blue arrow: Radial tear of the posterior horn of the lateral meniscus.

Preoperative MRI scans were analysed by one sports‐medicine trained orthopaedic consultant (L.W.) and concomitant injuries were recorded. Twenty patients have been analysed by a second sports‐medicine orthopaedic consultant (A.R.) to evaluate interrater correlation coefficient (ICC). The structures of interest were assessed on MRI using specific criteria. The KF were identified proximal to the lateral femoral condyle, adjacent to the branches of the superior lateral genicular artery in the sagittal and coronal plane [8]. Normal KF exhibited a uniform low signal with fibres running to their anatomical attachment at the distal femur. The ALC and the ALL were identified as a low‐signal structure originating posterior‐proximal to the lateral femoral epicondyle, crossing superficial to the fibular collateral ligament and deep to the ITB, and inserting on the tibia midway between Gerdy's tubercle and the fibular head (Figure 2). ALC and KF were classified as injured if: a visible discontinuity or bony avulsion were present, or indirect signs of injury like intrasubstance signal changes or a wavy appearance, focal bone marrow oedema at the femoral insertion or soft tissue oedema/haematoma in the region were noted.

Injuries to the lateral meniscus were analysed on MRI and confirmed during surgery. Tear type and location were also obtained on MRI and definitely defined during arthroscopy. A tear was classified as radial tear if a substantial part of meniscus was left at the root insertion and a side‐to‐side repair was possible. A root tear was defined as a direct avulsion of the tibial insertion where a transtibial fixation was necessary [13]. If a radial tear was present, a complete or incomplete disruption of the circumferential lateral meniscal fibres was documented during surgery. Additionally, MRI findings related to the medial compartment and medial soft tissue structures were documented.

### Statistics

Data were analysed using SPSS statistics software version 29.0 (IBM). An a priori sample size calculation analysis based on a recent study [28] was performed using G*Power v.3.1.9.7. A sample size of 114 patients (1:1 allocation) would detect the differences in means in ATLT of 0.4 ± 0.75 mm with a power of 0.81 and an alpha of 0.05 (effect size 0.53). Continuous variables are given as mean ± standard deviation, categorical variables as numbers and percentages. Normal distribution was analysed with Kolgomorov–Smirnov test. If normal distribution was confirmed, student's *t*‐test was used for comparison of means in continuous variables between patients with concomitant injuries to the KF, ALL and LM and with intact structures (i.e., SSD of ATLT in quantitative pivot shift). Analysis of variance with Bonferroni correction was used when more than two variables were tested (i.e., subgroup‐analysis of lateral meniscus tear types). Chi‐square test or Fisher's exact test was utilised to find differences in two nominal variables (i.e., clinical pivot shift classification). Pearson correlation test was used to identify correlations between two continuous variables. Binary logistic regression was used to calculate the odds ratio for having a high pivot‐shift. ICC was calculated with a two‐way mixed model for absolute agreement. Statistical significance was set at a value of *p* < 0.05.

## RESULTS

**Figure 3:**
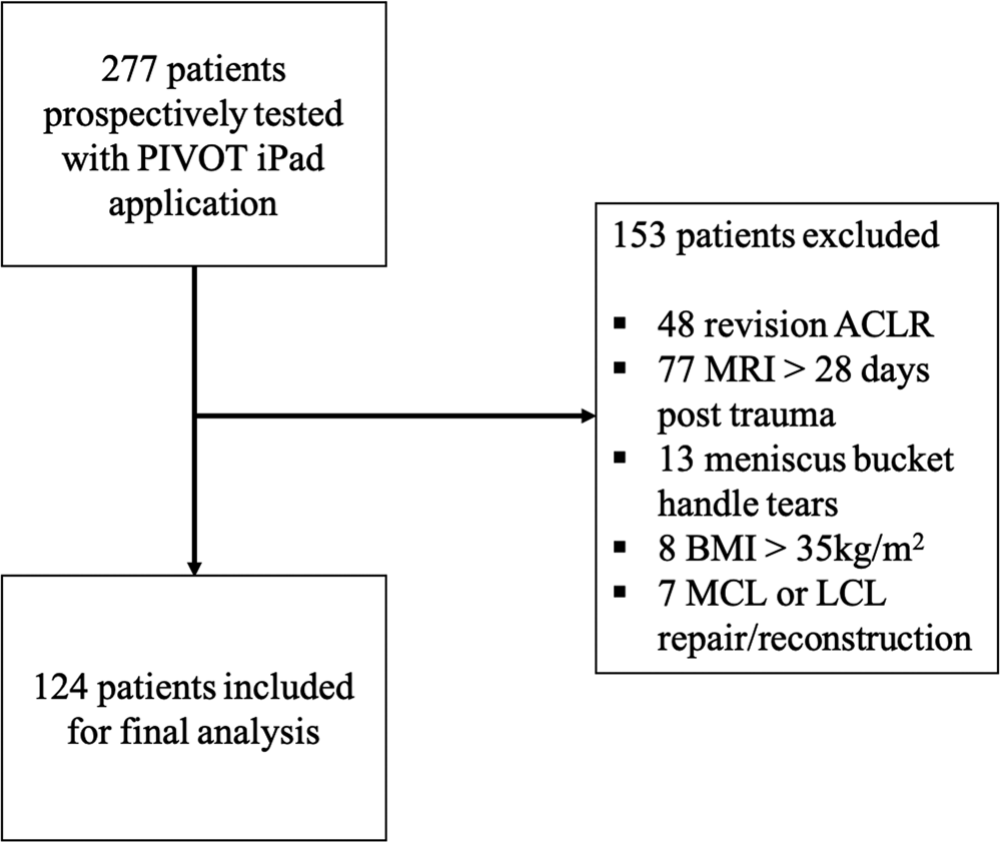
Flowchart of applying exclusion criteria on the entire patient cohort tested with the PIVOT iPad application.

A total of 124 patients were included for final analysis (Figure 3) and the patients' characteristics are listed in Table 1.

**Table 1:** Patients' characteristics.

Variable	Value
Age (years)	29.9 ± 11.2
Gender	76 (61%) men
	48 (39%) women
Laterality	76 (61%) right
	48 (39%) left
Body mass index (kg/m^2^)	24.7 ± 3.7
Injury mechanism	103 (83%) noncontact
	15 (12%) contact
	6 (5%) unknown
Interval trauma to magnetic resonance imaging	6.9 ± 6.6 days (range, 0–28 days)
Interval trauma to surgery	43.5 ± 32.2 days (range, 2–164 days)

### Prevalence of concomitant injuries

Injuries to the ALL occurred in 57 patients (48%) and to the KF in 51 patients (43%). A combined injury to the ALC and the KF was present in 33 (28%) patients. The lateral meniscus was injured in 47 (38%) patients, thereof 18 (15%) longitudinal, 14 (11%) radial, 11 complex (9%), 3 (2%) posterior root and 1 horizontal (1%) tears. Bucket‐handle tears were excluded for analysis. A combination of ALL, KF and LM tears was seen in 18 (15%) patients. The ICC between the two raters for injury to the ALL was 0.894 (95% confidence interval [CI] 0.741–0.957, *p* < 0.001) and for injuries to the KF was 0.946 (95% CI 0.867–0.979, *p* < 0.001).

### Pivot shift analysis

The quantified PS was significantly higher in the injured compared to the contralateral healthy knee (ATLT: 3.2 ± 1.6 mm vs. 1.5 ± 0.9 mm; *p* < 0.001).

Clinical IKDC pivot shift grading was assessed in 81 of 124 patients (65.3%). It was not recorded and documented in the first 43 patients. Of those, four patients exhibited a negative PS (grade 0), 40 patients PS grade I, 30 patients PS grade II and 7 patients PS grade III. Hence, 44 (54%) patients were classified as low‐grade and 37 (46%) patients as high‐grade PS. The high‐grade PS group showed a significantly higher ATLT compared to the low‐grade PS group (2.2 ± 1.8 mm vs. 1.3 ± 1.1 mm; *p* < 0.01).

Concomitant injuries to the KF, the ALL or the LM did not significantly increase the quantified PS. Also, combined injuries to the KF and ALL had no significant influence on the quantified PS. If all three structures were injured, the quantified PS was not significantly increased. Clinical IKDC PS grading showed a significantly higher proportion of high‐grade in patients with LM injury but not in patients with KF or ALL injuries (Table 2). Injury to the LM increased the risk of having a high‐grade PS by 2.5 (odds ratio; 95% CI 1.019–6.237, *p* < 0.05).

**Table 2:** The effect of concomitant injuries to the KF, ALC and the LM on preoperative quantified PS and IKDC pivot shift grading.

Concomitant injury		Mean SSD of quantitative PS (mm)	*p* value	IKDC Low‐grade PS (*N*, %)	IKDC High‐grade PS (*N*, %)	*p*‐value
KF	Intact (*N* = 70)	1.7 ± 1.6, range −0.7 to 6.7	*p* = 0.77	24 (29.6%)	21 (25.9%)	*p* = 1.0
	Injured (*N* = 54)	1.7 ± 1.3, range 0.0 to 5.9		20 (24.7%)	16 (19.8%)	
ALL	Intact (*N* = 64)	1.7 ± 1.5, range −0.3 to 6.7	*p* = 0.83	27 (33.3%)	19 (23.5%)	*p* = 0.37
	Injured (*N* = 60)	1.7 ± 1.5, range −0.7 to 5.9		17 (21.0%)	18 (22.2%)	
Combined KF + ALL	Intact (*N *= 88)	1.6 ± 1.4, range −0.7 to 6.7	*p* = 0.24	31 (38.3%)	24 (29.6%)	*p* = 0.63
	Injured (*N* = 36)	2.0 ± 1.5, range 0.1 to 5.9		13 (16.0%)	13 (16.0%)	
Lateral meniscus	Intact (*N *= 75)	1.7 ± 1.4, range –0.3 to 5.5	*p* = 0.99	30 (37.0%)	17 (21.0%)	*p* = 0.043
	Injured (*N* = 49)	1.7 ± 1.6, range −0.07 to 6.7		14 (17.3%)	20 (24.7%)	
Combined KF + ALL + LM	Intact (*N* = 103)	1.7 ± 1.5, range 0.7 to 6.7	*p* = 0.73	35 (43.2%)	28 (34.6%)	*p* = 0.69
	Injured (*N* = 21)	1.8 ± 1.7, range 0.5 to 5.9		9 (11.1%)	9 (11.1%)	

Abbreviations: ALC, anterolateral complex; IKDC, International Knee Documentation Committee; KF, Kaplan fibres; LM, lateral meniscus; PS, pivot shift; SSD, side‐to‐side difference.

An in‐depth analysis of lateral meniscus tear types revealed a significant difference in the quantitative pivot shift between complete radial tears and both intact menisci and all other types of meniscus tears (Table 3).

**Table 3:** The effect of different lateral meniscus tear types on preoperative ATLT during quantified pivot shift (124 patients) and IKDC pivot shift grading (81 patients).

Meniscus tear type	*N* (%)	SSD of ATLT (mm)	*p* value	IKDC low‐grade PS (*N*, %)	IKDC High‐grade PS (*N*, %)	*p* value
None	77 (62.1%)	1.7 ± 1.4, range −0.3 to 5.5		30 (62.5%)	18 (37.5%)	
Horizontal tears	1 (0.8%)	1.6		‐		‐
Longitudinal	18 (14.5%)	1.9 ± 2.0, range −0.7 to 6.7	*p* = 0.69	6 (46.2%)	7 (53.8%)	*p* = 0.35
Radial	14 (11.3%)	2.2 ± 1.5, range 0.7 to 5.9	*p* = 0.24	3 (30.0%)	7 (70.0%)	*p* = 0.08
– Incomplete radial	9 (7.3%)	1.5 ± 0.5, range 0.8 to 2.0		1 (16.7%)	5 (83.3%)	‐
– Complete radial	5 (4.0%)	3.5 ± 1.8, range 2.1 to 5.9	*p* = 0.027*	2 (50.0%)	2 (50.0%)	‐
			*p* = 0.012^#^			
Complex	11 (8.9%)	1.2 ± 0.9, range −0.2 to 3.0	*p* = 0.24	4 (57.1%)	3 (42.9%)	*p* = 1.0
Lateral posterior root avulsion	3 (2.4%)	0.9 ± 0.3, range 0.5 to 1.5	*p* = 0.10	1 (33.3%)	2 (66.7%)	*p* = 0.55

*Note*: Statistical significance compared to intact meniscus* and other meniscus tears^#^ (analysis of variance with Bonferroni correction). Abbreviations: ATLT, anterior translation of the lateral tibia; IKDC, International Knee Documentation Committee; *N* number; SSD, side‐to‐side difference.

### Confounding factors

Quantitative PS was not correlate with demographic factors such as age, height, weight or BMI of the patient (n.s.). Quantitative PS was not significantly different between male and female patients. The ATLT measured by quantitative PS was negatively correlated with the time from injury to MRI (*r* = −0.295; *p* > 0.01) but not with time between injury and surgery.

## DISCUSSION

The main findings of this study were twofold: first, a lateral meniscus tear was correlated with a clinically detected high‐grade PS; second, a complete radial meniscus tears of the lateral meniscus significantly increased the ATLT in the quantified PS test compared to patients with an intact meniscus or a different meniscal injury. Injuries to the ALL or to the KF were not associated with high‐grade PS or an increased ATLT. Even a combination of injury to the ALL and the KF was not associated with high‐grade PS.

ACL injuries rarely occur in isolation, with concomitant damage to peripheral structures and the menisci receiving growing attention within the orthopaedic community in recent years. Notably, injuries involving the KF, the ALL and the lateral meniscus have been extensively studied for their role in worsening knee instability [14, 26, 27, 46]. These investigations are critical for two main reasons. First, the prevalence of concomitant injuries is high, ranging from 19% to 85% for KFs [4, 6, 7], 51% to 76% for the ALL [10, 18, 37, 44] and 30% to 40% for LM tears [19, 34], and these injuries can often be reliably identified using preoperative MRI. Second, understanding the factors contributing to significant preoperative ALRI can improve treatment strategies and help prevent suboptimal clinical outcomes, persistent knee rotatory instability and ACL reconstruction failures. Thus, preoperative detection of significant injuries would allow surgeons to treat these injuries if necessary. Importantly, soft‐tissue injury detection is more accurate in timely acquired MRIs and diminishes from about 1 months after trauma [6, 17]. That is why we excluded patients with MRI acquisition over 28 days after injury.

Concurrent anterolateral surgical procedures, such as lateral extra‐articular tenodesis and ALL reconstruction, have been proposed to address severe ALRI [15]. These procedures are associated with reduced postoperative rotatory instability and lower re‐rupture rates, alongside favourable clinical outcomes and high rates of return to sports [16, 41]. However, clear indications for the addition of anterolateral stabilisation remain undefined [15]. Distinct structures on the lateral side of the knee including the anterolateral capsule, the ALL, and the femoral attachment of the iliotibial band (ITB), the KF, plays a significant role in ALRI [2]. In vitro studies have demonstrated that the KF are the primary restraint to tibial internal rotation beyond 30° of knee flexion [26, 46]. Several in vitro studies have reported only a minor increase in tibial internal rotation (1°–5°) when the ITB is sectioned alongside the ACL [14, 24, 46]. While KF injuries are therefore expected to contribute to increased ALRI, the extent of their impact on clinical knee laxity remains unclear. These subtle changes are often difficult to detect clinically using PS testing. Correspondingly, clinical studies have not consistently correlated KF injuries with increased PS grades [11, 43, 45]. Terry et al. identified a correlation between deep ITB injuries in ACL‐deficient knees and increased PS grades observed during physical examination [42]. Devitt et al. found no difference in IKDC PS grading between ACL‐injured patients with or without KF injuries, nor did KF injuries significantly increase quantitative PS measurements using electromagnetic systems [11]. The findings of this study align with previous research, as no significant differences were observed between study groups in either quantitative or clinical PS grading. It is important to notice that all detected injuries on MRI were included, not only high‐grade complete fibre disruptions.

The association between ALL injuries and ALRI remains similarly debated. While in vitro studies suggest that the ALL has a slight contribution to resisting internal tibial torque (10%–15% from 30° to 90° knee flexion) [26, 46], clinical studies present more pronounced effects. Several investigations have linked ALL injuries to higher preoperative PS grades [12, 34, 36, 40]. Song et al. found a higher prevalence of ALL injury (94%) in the high‐grade PS cohort compared to the low‐grade PS group [40]. Musahl et al. found a higher ALRI during quantified PS manoeuvre in patients with ALC injury [34]. Conversely, the study by Miyaji et al., found no significant difference in quantitative PS measurements between patients with and without ALL injuries [29]. Our results are consistent with the latter, as patients with concomitant ALL injuries did not exhibit increased ATLT and no correlation with higher PS grades.

Lateral meniscus injuries, particularly to the posterior horn, are also highly prevalent, with an incidence of 38% in the present study, consistent with prior reports [19, 34]. Although the lateral meniscus does not restrict internal tibial rotation in ACL‐intact knees [46], its injury or removal significantly increases rotational laxity [1, 32]. However, the clinical impact of lateral meniscus tears on PS measures remains inconclusive. Some studies have found associations between LM injuries and higher PS grades [23, 34, 35], while others did not [28]. Interestingly, in the present study, patients with LM tears exhibited clinically a high‐grade PS compared to patients with an intact meniscus. This finding was not confirmed for the ATLT measurement over the entire cohort. Yet, subgroup analysis revealed that complete disruption of the circumferential fibres (complete radial tears) significantly increased quantitative PS measurements, whereas tears with remaining circumferential fibres did not. This observation is limited by the small sample size of the subgroups and needs to be interpreted with caution. In contrary to previous studies [23, 35], LM root tears did not increase the ATLT significantly, which could be due to intact meniscofemoral ligament which maintained the ring structure. Lucidi et al. similarly reported no significant increase in quantitative PS in patients with posterior root tears of the lateral meniscus [28], though a detailed subgroup analysis of complete versus incomplete tears was not conducted.

In summary, this study adds new clinical evidence on the impact of concomitant injuries to the KFs, ALL and lateral meniscus on ALRI. However, a clear cause for a high‐grade clinical PS remains unpredictable. Based on these findings, a high‐grade PS cannot simply be explained by the presence of concurrent injuries but several other patient‐specific factors including tibial and femoral bony and soft tissue morphology, joint hyperlaxity or type of injury need to be considered when ALRI is assessed. Further high‐quality clinical studies are necessary to better define the indications for anterolateral stabilisation and its role in managing concomitant injuries in ACL‐injured knees.

This study has some limitations. MRI scans were performed by different institutes with different quality, as this represents the common daily practice. The PS testing was performed by three different investigators. Even though all were thought prior to testing, there might be an intra‐investigator difference in performing the PS manoeuvre. Analysis of clinical IKDC PS grading was only performed in 81 patients as it was not documented in the first 43 patients. Potential confounding factors such as hyperlaxity or bony morphology was not included in the analysis [5]. An a priori sample size calculation was based on a SD of ±0.75 mm, which is smaller than the actual SD of the groups and could result in an underpowered analysis. However, the differences in means between the groups were comparable and not deemed clinically significant.

## CONCLUSION

In this cohort of ACL‐injured knees, concomitant injuries to peripheral anterolateral soft‐tissue structures did not significantly increase the quantitative pivot shift in this cohort of ACL‐injured knees. Even combined injuries to those structures had no significant effect on anterior tibia lateral translation (ATLT). Clinical PS grading was higher in patients with lateral meniscus injury and ATLT during PS manoeuvre was significantly increased in patients with a complete disruption of the circumferential meniscus fibres (complete radial tear). However, limited power may have reduced the ability to detect a true effect and a type II error cannot be ruled out.

## AUTHOR CONTRIBUTIONS

All authors contributed to the study conception and design. Lukas Willinger, Armin Runer and Philipp Winkler performed material preparation, data collection and analysis. The first draft of the manuscript was written by Lukas Willinger, and all authors commented on previous versions of the manuscript. Philipp Winkler and Sebastian Siebenlist provided critical revision and final approval of the version to be published. All authors read and approved the final manuscript.

## CONFLICT OF INTEREST STATEMENT

Sebastian Siebenlist is a consultant for Arthrex GmbH, medi GmbH and Co KG and KLS Martin Group. Julian Mehl is a consultant for Arthrex GmbH and Ormed GmbH, Andrea Achtnich is a consultant for Arthrex GmbH. The remaining authors declare no conflicts of interest.

## ETHICS STATEMENT

Ethics Committee of the Technical University of Munich (No: 198/21 S‐KK). All patient gave their informed consent to participate in this study.

## Data Availability

The data that support the findings of this study are available from the corresponding author upon reasonable request.
